# Approaches and strategies used in the training and supervision of Health Extension Workers (HEWs) delivering integrated community case management (iCCM) of childhood illness in Ethiopia: a qualitative rapid appraisal

**DOI:** 10.4314/ahs.v18i1.24

**Published:** 2018-03

**Authors:** Duduzile Nsibande, Marian Loveday, Karen Daniels, David Sanders, Tanya Doherty, Wanga Zembe

**Affiliations:** 1 Health Systems Research Unit, South African Medical Research Council, Cape Town, South Africa; 2 School of Public Health, University of the Western Cape, Bellville, South Africa; 3 School of Public Health, University of Witwatersrand, South Africa; 4 School of Child and Adolescent Health, Faculty of Health Sciences, University of Cape Town, Rondebosch, South Africa

**Keywords:** Community health workers, health extension workers, integrated community case management, training, supervision, Ethiopia

## Abstract

**Background:**

Globally, preventable and treatable childhood conditions such as pneumonia, diarrhoea, malaria, malnutrition and newborn conditions still account for 75% of under-five mortality. To reduce the mortality rate from these conditions, Ethiopia launched an ambitious Health Extension Programme (HEP) in 2003. Trained Community Health Workers (CHWs), named Health Extension Workers (HEWs) were deployed to deliver a package of care which includes integrated Community Case-Management (iCCM) of common childhood diseases.

**Objectives:**

This qualitative study aimed to explore approaches and strategies used in the HEW training and supervision as part of an evaluation of the Catalytic Initiative to Save a Million Lives.

**Method:**

A qualitative rapid appraisal study using focus group discussions and in-depth interviews was conducted.

**Results:**

Training of HEWs followed a cascaded training of trainer approach supported by implementing partners under guidance of the Ministry of Health. A comprehensive planning phase enabled good coverage of districts and consistency in training approaches. Training was complemented by on-going supportive supervision. HEW motivation was enhanced through regular review meetings and opportunities for career progression.

**Conclusion:**

These findings describe a thorough approach to training and supervision of HEWs delivering iCCM in rural Ethiopia. Ongoing investments by partners will be critical for long-term sustainability.

## Introduction

Despite the progress made in reducing under-five mortality, 75% of deaths globally are still caused by a few preventable and treatable conditions, namely pneumonia, diarrhoea, malaria, malnutrition and newborn conditions^1^,^2^. The 2014 UN Inter-agency Group for Child Mortality Estimation (IGME) report suggests that children in sub-Saharan Africa (SSA) and South Asia face a higher risk of dying before their fifth birthday^3^. Although effective treatments for common childhood conditions exist, the coverage for high-impact interventions remains unacceptably low and varies across population groups^4^,^5^.

The critical shortage of human resource is a major bottleneck in scaling up life-saving interventions especially in low-income countries. Task-shifting, using trained community health workers (CHWs), has been recognised as a viable strategy to complement and support services of other health workers in order to accelerate coverage and improve access to basic health services for underserved rural populations^6^,^7^. With training and supportive supervision, CHWs can deliver a package of simple maternal and child health interventions^8^,^9^.

**Figure 1:**
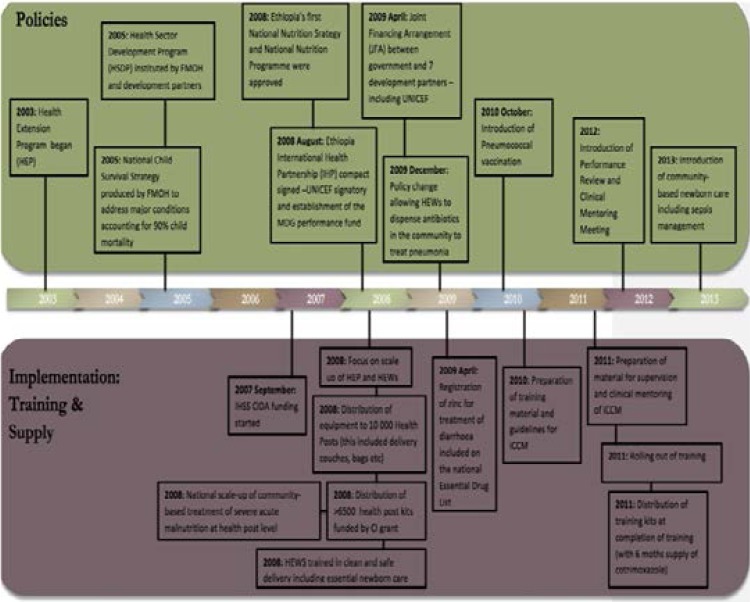
Health Reforms in Ethiopia (2003–2013)

Between 2003 and 2013 the Ethiopian Federal Ministry of Health (FMOH) was involved in major health reforms through policy reform, training and human resource distribution^10^. Some of these policies included the introduction of the Health Extension programme (HEP), the Health Sector Development Plan (HSDP), the first National Nutrition Strategy, the Joint Financial Arrangement (JFA) and allowing Health Extension Workers (HEWS) to dispense antibiotics in the community (Figure 1). The HEP included the construction of health posts, training and supervision of HEWs and the development of supervisory tools^11^,^12^.

The HEP is a flagship programme launched as a vehicle to achieve the MDGs 4 and 5 targets (to reduce under 5 child mortality by two thirds and maternal mortality by three-quarters between 1990 and 2015 respectively). One component of the HEP, which was scaled up in 2009, is the provision of integrated community case management (iCCM) of malaria, pneumonia and diarrhoea by HEWs at health posts^13^. By 2010, Ethiopia had the largest iCCM programme in Africa^14^. Several studies conducted in Ethiopia have confirmed that the iCCM programme has been successfully scaled-up and that community-based HEWs can correctly manage multiple treatable childhood illnesses^14^–^16^.

In Ethiopia, neonatal mortality rate (NMR) has remained unchanged and still accounts for 42% of all U5 deaths^17^. However, Ethiopia managed to reduce the under-five mortality rate (U5MR) by 28% between 2005 and 2011^3^,^18^. According to the 2014 IGME estimate, Ethiopia achieved the target for Millennium Development Goal 4 for child survival^3^.

In this paper, we describe stakeholder views of approaches and strategies used in the training, supervision and clinical mentoring of HEWs delivering iCCM in Ethiopia using a qualitative rapid appraisal design.

## Methods

### Study context

Ethiopia is the second-most populous (91.7 million) and one of the least urbanized nations on the African continent^19^. Over the past decade, different categories of CHWs have been trained in Ethiopia: traditional birth attendants (TBAs), community based reproductive health agents (CBRHAs), community health agents (CHAs) and Health Extension Workers (HEWs).

### Study design

This descriptive qualitative^20^ study by rapid appraisal^21^ was part of an external multi-country evaluation of UNICEF's Catalytic initiative (CI), Integrated Health System Strengthening (IHSS) program in 6 countries in Africa: Ethiopia, Malawi, Ghana, Mozambique, Niger and Mali^22^. In Ethiopia, the IHSS program provided support to five of ten regions in Ethiopia (Amhara, Benshangul, Oromia, SNNPR and Tigray)^22^. The rapid appraisal approach uses a less structured format for data collection in order to obtain the required information in a timely and cost-effective manner^21^.

### Data collection

Qualitative data was gathered through rapid appraisal^21^ during country field work and through a desk review of relevant documents (annual project reports from UNICEF and implementing partners, annual country reports to UNICEF, national strategic plans and academic published literature). A field visit of 10 days, by a mixed skill team of 3 researchers, took place during October 2012. Potential organisations and individuals for key informant interviews and focus group discussions (FGDs) were identified through a desk review process and were shared and amended in collaboration with UNICEF headquarters (HQ) and the Ethiopia country office.

Semi-structured interview guides were developed for each category of respondent (Ministry of Health, implementing partners, district management team, facility-based health workers and HEWs). These guides were reviewed by colleagues in UNICEF to ensure that they sufficiently captured the critical themes of the evaluation.

Each semi-structured interview was conducted by one or more researchers at the work places of the interviewees and interviews lasted between 30 minutes and one hour. Where necessary, especially with HEWs, a translator was used. Interviews were audio-recorded after permission was granted, and researchers took notes.

The country visit included 3 days of meetings with UNICEF, Ministry of Health, IPs and other national-level stakeholders in Addis Ababa, followed by travel to outlying districts for visits to district management teams and health facilities. The researchers visited Gojam and Gondor districts in the Amhara region and Shewa district in Addis Ababa. The selection of districts was made in consultation with the UNICEF country office taking into consideration travel distances, time constraints and occurrence of important activities relevant to the evaluation. The field visit was planned to coincide with the 14^th^ Health Sector Annual Review meeting taking place in Bahir Dar, the capital of the Amhara region. This enabled the researchers to interview a wide range of stakeholders from national, regional and district levels in order to gain a composite picture on which to base the findings. We also visited health posts and health centers in order to observe and validate data collected during interviews and to gain an understanding of the HEW programme. Key informants included officials from the UNICEF country office, Ethiopian Federal Ministry of Health, Implementing partners (IPs), Nurses, HEWs and their supervisors (Table 1).

**Table 1:** Summary of participants

	Participant category	Number of interviews
**Individual interviews**	Federal Ministry of Health	12 males, 8 females
	Partners and researchers (P/R)	9 male, 7 female
	Nursing staff — includes midwives, community health nurses, community health nurses, enrolled nurses, registered nurses (N)	3 males, 4 females
	UNICEFCountry office UNICEF Regional office WHO	2 males, 2 females 2 males 1 respondent
**Focus Groups**	HEWs (2–8 per group) Health Development Army (HDA)	17 females 2 males, 4 females

### Data analysis

Four researchers (DN, ML, WZ and KD) independently reviewed the transcripts typed from the digitally recorded data. We conducted a simple manifest analysis of the qualitative material^20^,^23^. Since this was not an ethnographic study we were simply interested in what happened and what was experienced rather than trying to understand the deeper meaning of the experience. Exploring such meaning was not our evaluation intention and would have required a different study design. The four researchers annotated our reflections while reading, and then came together to discuss, compare and critique our insights. Based on this analysis the data was electronically (using a word processor) grouped into categories, the results of which are reported in narrative form in this paper.

### Ethical considerations

Ethical approval for the study was obtained from the South African Medical Research Council (EC026-9/2012) and approval was obtained from the Ethiopian Federal Ministry of Health (FMoH) and UNICEF Ethiopia country office. Interviewees were given detailed explanation regarding the purpose of the interview and their rights, including the right not to participate. Signed informed consent from literate participants and recorded verbal consent from illiterate participants was obtained by the interviewer.

## Results

### Preparation for the delivery of iCCM

#### Collaborative planning phase

Key informants confirmed that the iCCM preparation phase lasted for almost two years. We learned from informants attending the Health Sector Annual Review meeting that the iCCM design process was a collaborative effort between the FMoH, UNICEF and implementing partners. This collaboration resulted in the establishment of stakeholders' forums; namely the Technical Working Groups (TWG) at national and regional levels and Child Survival Working Groups to support and guide iCCM implementation. In-depth interviews revealed that these forums were responsible for developing iCCM integrated plans, standardized training materials, job aids, and monitoring and evaluation frameworks and tools.

“*National coordination of all Partners including Government saw a harmonised and well supported programme being implemented.... Partnership was strengthened through these coordinated activities at the National Level involving all partners with no claim of individual ownership. Communication across partners was key*”. [Implementing partner A]

“*Meetings are monthly, quarterly, biannually and so on. And you know, the most important thing, when this review meeting is done, the Ministry of Health is chairing it, usually. Even the Child Survival Technical Working Group at regional level is coordinated by the regional health, and UNICEF staff is the secretary*.” [Implementing partner B]

Key informants told us that part of the preparation included extensive work with implementing partners to ensure that all those who were going to be conducting the training of HEWs were involved in the development of training materials to ensure standardization.

“*So UNICEF has done a marvellous job in terms of coordinating us; different partners who are working in iCCM. They create forums where our partners come together and again share experiences.*” [Implementing Partner C]

The Ethiopia FMoH was described as supportive of the iCCM programme, ensuring that training activities were institutionalized within government programmes.

“*The government now gives priority to maternal, child and newborn health. So you know, this iCCM was introduced at the appropriate time actually, when the government is really serious about reducing maternal and child mortality and, you know, for achieving MDGs*.” [Implementing partner C]

### The role of Implementing partners in supporting iCCM training initiatives

Informants explained that different funding streams (CIDA funding, the MDG pool fund and other strategic commitments from the Ministry of Health and development partners) supported the training, supervision and mentoring in different *woredas* (districts).

“*The major broker was the Government driven by the desire to meet MDGs. Funding of our work is largely from USAID and UNICEF, through the catalytic Initiative is supporting the “logistics”, training materials and kits. The rest is funded from USAID. The programme is harmonised with government policy and programmes for iCCM. Government led, and different Partners assumed responsibilities for different areas in the country which ensured no overlap and therefore duplication*”.[Implementing partner A]

“*UNICEF and the implementing partners fund the planning and the co-ordination of the Performance Review Clinical Monitoring meetings*.”[FMoH Official C]

“*We cover 292 districts, in all those districts we took a mandate of covering the training technically [and] financially. Our own staff facilitated the trainings and also we covered the expenses but the logistics part is totally covered by UNICEF. We collaborated with UNICEF for the logistics for the drug supply.*” [Implementing partner B]

“*(Our organization) is involved in the other non-iCCM activities e.g. EPI, and trainings around common childhood illnesses in the target districts...No problems because of the strong partnership across key stakeholders. HEWs were trained properly by TOTs, and also frontline workers*.” [Implementing partner A]

Informants confirmed during interviews that different implementing partners conducted training in the districts where they operate to avoid overlaps and the duplication of services.

“*…for example L10K came up with a list of areas to cover and [the] same applied with the IFHP [USAID funded Integrated Family Health Programme]and Save the Children. In order to avoid overlapping of areas and duplication, the organisations agreed on the areas they each would work in. L10K was given 113 districts*.” [Implementing Partner B]

We also learned that although the training programme was standardised, it allowed some flexibility with regard to entry criteria. For example, in some pastoral communities, the requirement to have completed high school was waived:

“*In some areas, they don't only recruit people that have finished grade 10. The criteria is set by the government. So if you want to get grade 10 women only in Afar or Somali it is very difficult*.” [Implementing Partner D]

### Training strategy and content

We learned from discussions with key informants that HEW training adopted a train-the trainer (cascaded) approach. This was rolled -out through a7-day training of a pool of master trainers from the FMOH, implementing partners and universities at national level, who then cascaded training to regions and *woredas*.

**Figure 2:**
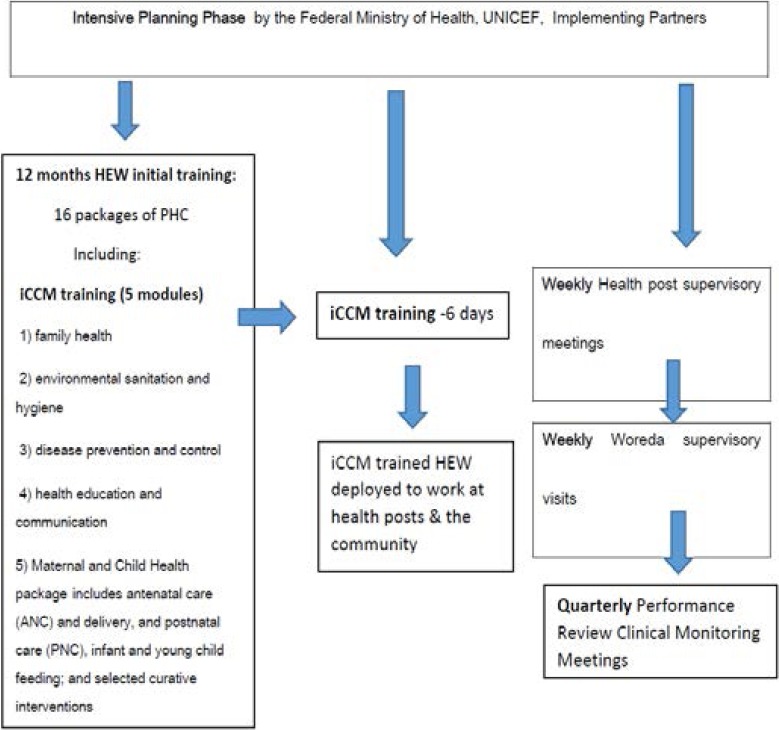
HEWs training and supervision

Overall, HEW training consisted of sixteen packages of primary health care (PHC) covered during the first 12 months. iCCM covered 5 modules which included family health, environmental sanitation and hygiene, disease prevention and control, health education and communication, Maternal and Child Health package (including antenatal care (ANC) and delivery, and postnatal care) and infant and young child feeding; and selected curative interventions (figure 2).

“*A sub-committee of the iCCM group was responsible for revising the materials, Trainer of Trainers, and provided oversight on cascading down the training to zonal offices, and the HEWs, and their supervisors*”. [Implementing Partner A]

“*Most of the government staff were trained as trainers who then trained the HEWs*.” [Implementing Partner C]

Thereafter HEWs were expected to attend the 10-day refresher training course focusing on clean delivery so that they could provide safe deliveries of babies in the community. Training tools such as videos, CDs, DVDs and child booklets, were available to support HEWs to render good quality health care.

### Supervision approach

According to key informants, HEWs receive clinical mentoring conducted by health centre staff and structured supervision in the form of review meetings which occur 4–6 weeks after initial training. During these meetings, iCCM registers are reviewed to assess data skills, caseloads and clinical performance. Supervision at health post is a collaborative effort between *woreda* and zonal focal health persons, implementing partners and health centre supervisors, as key informants described:

“*We also received training for one day to get supervision skills on the last day of the 7 day training to prepare us for follow-up at health posts*”. (*Woreda* Supervisor)

“*Supervisory sessions at woreda cluster level include structured supportive supervision (of HEWs) every 4 to 6 weeks by the trained health centre staff/supervisor, quarterly review meetings at the district level looking at all issues relating to the health problems/diseases and gaps that might exist*” (*Woreda* Supervisor ).

“*Supervision is comprehensive and covers all primary health care aspects. We look at registers for completeness, case management, classification, cold chain management recording*” (*Woreda* Supervisor)

“*iCCM has contributed to the broader national Child Survival Strategy and Plan. iCCM has led to positive spill-over effects for PHC in general in that resources used for iCCM supervision have led to the improvement of other programs (because) the iCCM approach is integrated supervision*”. (Implementing Partner B).

### HEW career development

FGDs with HEWs attending the 14^th^ Health Sector Annual Review meeting revealed that they felt that their career structure had been improved, in addition to their receiving routine training. They stated that attending the annual meeting made them feel their contribution was recognised and it helped to broaden their knowledge of other related health programmes. They also expressed that being salaried government workers improved their motivation and performance.

“*No one (HEW) has resigned since we started working (from my health post) in the last 12 months. But 2 (HEWS) left from other HPs because they got better jobs as traders. Yes, it was due to lack of benefits as HEWs. There was no career development; no upward movement even when you work for many years, but the government has started career development now*.” [Health Extension Worker]

Key informants explained that the FMoH was in the process of developing a defined career structure for HEWs and upgrading their career path. This change was appreciated by most informants and they felt it brought about a sense of job satisfaction among HEWs, minimised staff attrition and further contributed to the success of the programme.

“*We are now considering level 3 and level 4 training for HEWs which will be a diploma. A thousand HEWs are already enrolled and this will increase to 3000. iCCM training will continue until all HEWs reach level 4. Guidelines on transfer of HEWs are being developed and the plan is to include the HEP as part of pre-service training*.” [FMoH official 1]

## Discussion

This qualitative rapid appraisal describes the views of key stakeholders on strategies and approaches to training and supervision of HEWs within the iCCM programme in Ethiopia. Similar to other studies^24^,^25^, our findings suggest that government leadership, strong political will and stakeholder collaboration helped to create an enabling environment which facilitated training and supervision activities.

Importantly, even though most iCCM activities were standardized, the flexibility given to implementing partners in how they trained and facilitated training updates ensured that it was adapted to local contexts. The continued scale up of Ethiopia's iCCM programme hinges on its prioritization, intensive planning and budgeting as well as coordinated training and supervision^26^. On-going training and supervision of Ethiopian HEWs results in the improvement of their knowledge and clinical skills. Other studies on iCCM supervision in Ethiopia have reported an improvement in the classification and management of children under-5 years between pre and post-test evaluations of HEW performance^15^,^26^,^27^. Furthermore, integrated supervision allows healthy cross-fertilization of ideas between different programmes which has a positive spill-over effect to other primary health care programmes. These study findings are consistent with other studies conducted in Ethiopia and elsewhere which showed that when CHWs are trained and provided with adequate resources within a structured supervised environment, they can successfully manage common childhood illnesses within their communities^15^,^16^.

Health worker attrition has serious implications for the effectiveness of maternal and child health programmes^28^,^29^. Ethiopia has reduced HEW attrition by ensuring that they are salaried government workers entrenched in the health system. Our findings also suggest that improvements in HEW career structure have contributed positively to their motivation and retention.

However, there needs to be a balance between formalised career pathways for CHWs and their community change agent role. A major focus for CHWs in the Alma Ata Declaration was community involvement in prevention and promotion, but due to several factors, including the challenge of the health Millennium Development Goals, there has been a shift in sub-Saharan countries towards using CHWs to increase access to treatment^30^. In many countries, CHWs were previously recruited (often drawing from a pool of volunteers operating at village level) and provided with basic health training to deliver various health promotion and prevention activities. With the increased momentum towards child survival goals in sub-Saharan Africa, this tier of CHWs has increasingly taken on curative functions. The discourse has been one of ‘task shifting’ within primary health care, from clinic level, and formal clinicians, to CHWs at health posts^6^. As CHWs become more formalised members of the health workforce with increasing curative functions, there is a risk that their community mobilisation and action role is neglected and yet this role remains critical given the ongoing contribution of social determinants in child health^31^.

Whilst this study has found that the FMoH has provided strong leadership in guiding the training and supervision of HEWs, financing of these activities has largely been provided by implementing partners. Over the past decade, Ethiopia has received considerable official development assistance (ODA) for maternal, newborn and child health (MNCH) and has successfully guided partner support towards the health sector development programme enabling joint financing to ensure implementation of government policies and plans^32^. The annual MNCH ODA has increased from $105 million in 2003 to $215 million in 2010^33^. As a result, external resources for health, as a percentage of total health expenditure, increased from 16% in 2000 to 52% in 2011^19^. This situation has implications for the sustainability of the activities described in this paper which require ongoing budgetary prioritisation and investment.

## Limitations

This was a rapid appraisal study which did not cover all implementing regions. Thus the impressions presented must be regarded as a snapshot, raising questions for further exploration, particularly regarding the impact of the identified strategies on HEW performance and quality of care. Our research could have benefited from more time in the field to allow for deeper exploration of the experiences of the training strategies and to conduct observation during training and supervisory meetings. Despite these limitations, this qualitative study has provided valuable descriptions of strategies adopted for training and supervising HEWs which could provide lessons for other countries scaling up iCCM.

## Conclusion

This study shows that investing in on-going training and supportive-supervision of HEWs has contributed to motivation of HEWs in rural Ethiopia. Overall, these results outline a picture of a well-structured training and supervision HEW programme coordinated by the Ethiopia Federal Ministry of Health with well co-ordinated support from implementing partners.
